# OrthoFusion: A Super-Resolution Algorithm to Fuse Orthogonal CT Volumes

**DOI:** 10.21203/rs.3.rs-4117386/v1

**Published:** 2024-04-03

**Authors:** Rebecca E. Abbott, Alain Nishimwe, Hadi Wiputra, Ryan E. Breighner, Arin M. Ellingson

**Affiliations:** University of Minnesota; University of Minnesota; University of Minnesota; Hospital for Special Surgery; University of Minnesota

**Keywords:** Super Resolution, Bone Models, Computed Tomography, Image Fusion, Spatial Resolution Enhancement

## Abstract

OrthoFusion, an intuitive super-resolution algorithm, is presented in this study to enhance the spatial resolution of clinical CT volumes. The efficacy of OrthoFusion is evaluated, relative to high-resolution CT volumes (ground truth), by assessing image volume and derived bone morphological similarity, as well as its performance in specific applications in 2D-3D registration tasks. Results demonstrate that OrthoFusion significantly reduced segmentation time, while improving structural similarity of bone images and relative accuracy of derived bone model geometries. Moreover, it proved beneficial in the context of biplane videoradiography, enhancing the similarity of digitally reconstructed radiographs to radiographic images and improving the accuracy of relative bony kinematics. OrthoFusion’s simplicity, ease of implementation, and generalizability make it a valuable tool for researchers and clinicians seeking high spatial resolution from existing clinical CT data. This study opens new avenues for retrospectively utilizing clinical images for research and advanced clinical purposes, while reducing the need for additional scans, mitigating associated costs and radiation exposure.

## INTRODUCTION

Three-dimensional visualization and creation of bone models are essential for many applications in medical imaging, such as diagnosis of fracture or disease, pre-operative planning, implant design, trainee and patient education, and motion analyses requiring digitally reconstructed radiographs (DRRs). Computed tomography (CT) is a common imaging modality that uses x-rays to produce cross-sectional images of the body, offering detailed volumetric renderings of anatomy and enabling precise examination of internal structures. Although CT is the gold standard for morphologic bone imaging and thus generation of bone models, CT acquisition incurs radiation exposure, cost, and time. Thus, there are concerted efforts across disciplines to reduce radiation exposure to patients to be ‘as low as reasonably achievable’ (ALARA) for the clinical indication. Despite these risks, clinical CT orders are still increasing.^1^

Clinical CT scans are routinely archived in the patient’s electronic medical records (EMR). Typical clinical scans in EMR are highly anisotropic for a variety of reasons, but primarily driven by methods to reduce both radiation exposure to patients and image file size to account for storage limitations. Imaging protocols export 2 or 3 orthogonal volumes (axial, sagittal, and/or coronal) with high in-plane resolution (e.g., 0.3×0.3 mm), but with greater slice thickness and spacing, resulting in a 10-fold courser through-plane resolution (e.g., 3 mm). Unfortunately, high spatial resolution and cortical bone contrast are necessary for visualization of the segmentation process necessary for bone model construction. Therefore, spatial resolution of many CT volumes in EMR is inadequate for multiplanar reconstruction and bone modelling applications.

A method to increase the spatial resolution of clinical CT volumes obtained from EMR would open new opportunities for researchers and clinicians to create multi-planar reconstructions and accurate bone models from the wealth of existing data already stored in EMRs. Super-Resolution is a broad term that refers to a family of techniques to combine multiple low-resolution volumes to produce higher resolution volumes.^2–4^ Various super-resolution techniques have been employed to improve medical imaging quality and resolution, including iterative reconstruction^2^, deconvolution^3^, deep learning-based approaches^5^, and registration and fusion^6^. ‘Registration and fusion’ involves spatially aligning multiple volumes (registration) and combining the information to a single volume that incorporates data from multiple views (fusion). Despite these technological advancements, super-resolution tools in medical imaging are often tailored to a specific research task and are not readily available to researchers or clinicians, requiring advanced programming skills to implement. Additionally, many require large datasets for learning or are not generalizable to other applications.

The objective of this study was to design, implement, and evaluate an intuitive super-resolution algorithm to increase the spatial resolution of low-resolution clinical CT volumes. This effort was motivated by the fact that some participants in our cervical kinematics studies already had recently acquired clinical CT imaging in their EMR. To avoid an additional CT scan and associated radiation exposure, an ethical alternative was desired. Our application requires a subject-specific bone model created from a CT volume to build the DRR for shape-matching via biplane videoradiography. Biplane videoradiography is an advanced imaging technique that captures dynamic radiographs from two views simultaneously so that 3-D bone kinematics can quantified. Our super-resolution solution is generalizable beyond our specific application and will provide an opportunity to other researchers and clinicians to use clinical CTs from EMR to create high-resolution 3-D visualizations and bone models.

## METHODS

### OrthoFusion Super-Resolution Algorithm

**Figure 1:**
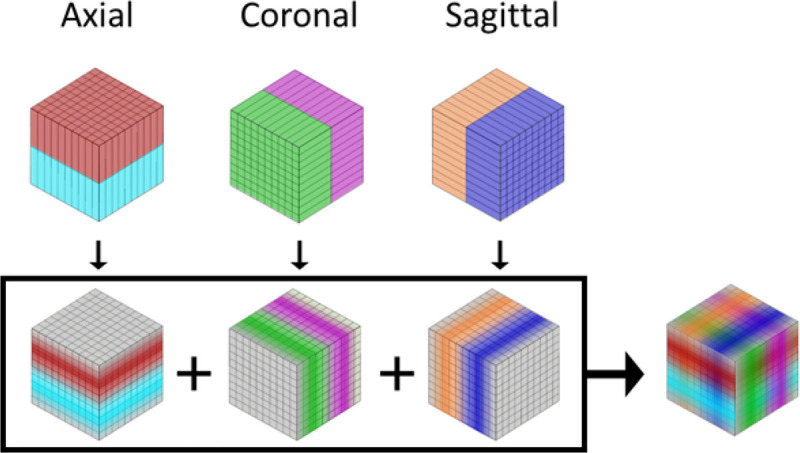
OrthoFusion Super Resolution Algorithm. Orthogonal clinical volumes with low through-plane resolution (top row) are linearly interpolated onto an isotropic high-resolution 3-D grid (bottom row). A voxel-by-voxel average is computed to produce the super resolution volume (right).

The OrthoFusion super-resolution algorithm registers and fuses 2 or 3 orthogonal low-resolution CT volumes to create a single volume with high spatial resolution. A graphical representation of the algorithm is provided in Fig. 1, starting from the axial, coronal, and sagittal “Clinical” volumes. First, each low-resolution clinical volume is linearly interpolated to the same high-resolution isotropic grid (0.2 × 0.2 × 0.2 mm) such that an intensity value is assigned to each voxel (registration). Second, the voxel-by-voxel average is computed (fusion). By including multiple orthogonal volumes (axial, coronal, and sagittal), all available information is utilized, capturing features that would otherwise be missed.

### Image Acquisition and Processing

To evaluate the effectiveness of the OrthoFusion algorithm, we acquired high-resolution CT volumes to serve as the ground truth. CT imaging (B60S; Siemens Biograph PET/CT, Knoxville, USA) of the cervical spine was acquired from 4 participants (3 female/1 male, age 21 to 40 years old) as part of a larger study approved by the University of Minnesota Institutional Review Board. Participants provided informed signed consent and all methods were performed in accordance with all relevant guidelines and regulations. The CT scans were reconstructed into 3 orthogonal high-resolution volumes (axial, sagittal, and coronal), with in-plane resolutions of approximately 0.2 × 0.2 and a through-plane resolution of 0.6 mm.

### Image Processing

**Figure 2:**
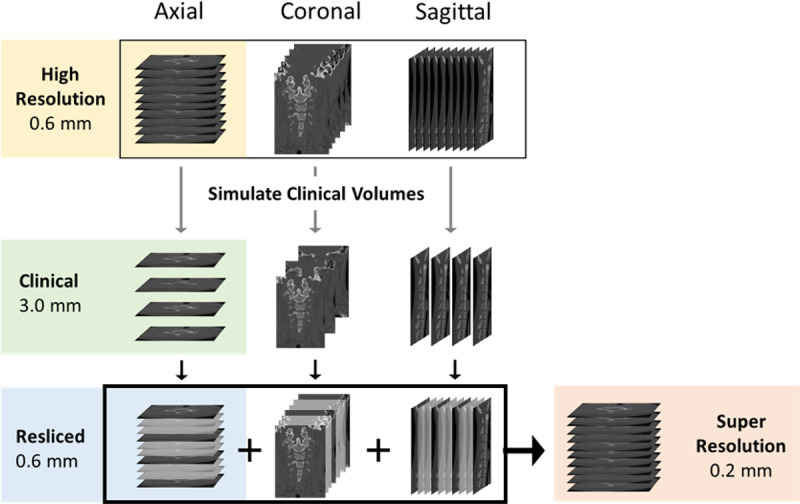
Study design. Four cases were extracted to evaluate the OrthoFusion algorithm – High Resolution (HR), Clinical (Clin), Resliced (RS), and Super Resolution (SR). The axial HR volume was considered the ‘ground truth’. First, HR CT volumes were acquired and reconstructed into 3 orthogonal volumes (axial, coronal, sagittal) with in-plane resolutions of ~0.2 × 0.2 mm and a through-plane resolutions of 0.6 mm (top row). Next, Clin volumes were simulated by down-sampling the HR volumes using Gaussian interpolation to a 3 mm through-plane resolution (middle row). The OrthoFusion algorithm (box) was applied, as depicted in Figure 1. The RS case volume was derived from the axial Clin volume by interpolating back to a 0.6 mm through-plane resolution using Lanczos interpolation.

Four CT cases were compared within each participant: 1) high-resolution (HR), 2) clinical (Clin), 3) resliced (RS), and 4) super-resolution (OrthoFusion) volumes. Image processing was performed using custom code in MeVisLab 3.7.2 (MeVisLab Medical Solutions AG). The study design is illustrated in Fig. 2. An axial clinical (Clin) volume with a through-plane resolution of 3 mm was simulated by down-sampling the axial HR volume using Gaussian interpolation. A resampled (RS) volume was created by then up-sampling the axial Clin volume back to 0.6 mm through-plane resolution using Lanczos interpolation, a commonly used technique.^7^ The super-resolution (OrthoFusion) volume was constructed using the OrthoFusion algorithm described above, fusing all 3 orthogonal volumes and resulting in an isotopic resolution of 0.2 mm.

### General Procedure

#### Bone Segmentation

Following image acquisition and processing, subject-specific bone models of the C4 and C5 vertebrae were segmented from each CT case in Mimics (v23, Materialize, Leuven, Belgium) by RA – an experienced segmentation expert. Initial segmentation was performed using a semi-automated CT bone segmentation tool (CT Bone Wizard) that utilizes initial thresholding and a continuity-based algorithm. Additional manual refinement was performed as needed. The segmented CT volumes were then used to create 3-D geometries for morphological comparisons and Digitally Reconstructed Radiographs (DRRs) for kinematic analysis.

#### Segmentation Time

The approximate time and tools (semi-automated vs. manual) required for segmenting each cervical vertebra were recorded for each case.

#### Image Similarity

Image quality assessment was performed comparing each case to the respective HR volume using the structural similarity (SSIM) index and peak signal to noise ratio (*PSNR*).^8^ The structural similarity map includes each pixel in the image, based on its relationship to other pixels in a local radius. The measures were calculated on masked CT volumes to isolate the bone, and on the whole CT volumes. The mean and standard deviation of the local *SSIM* values were compared between cases. The *PSNR* was computed on the whole volumes.

#### Morphological Similarity

The morphological similarity of the subject specific 3-D bone geometries was assessed using CloudCompare (v2.10). The point-by-point 3-D minimum Euclidean distance between the case geometries and reference HR geometry was computed to determine 1) bias: the mean signed error, 2) precision: the standard deviation of the signed error, and 3) MAE: the mean absolute error.

#### Application Specific Procedure

**Figure 3:**
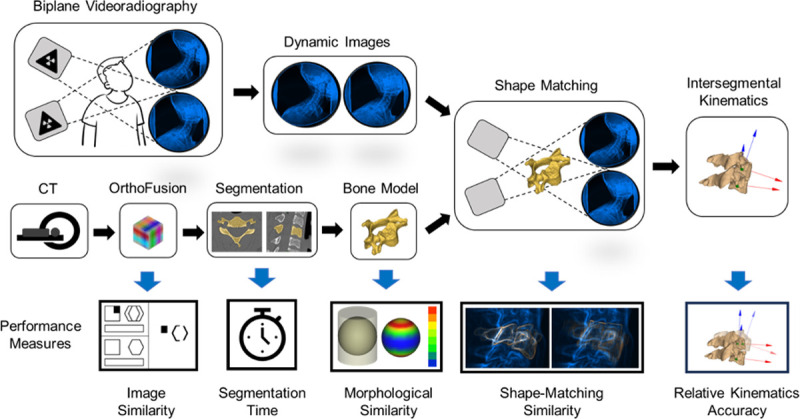
The biplane videoradiography procedure for measuring intersegmental kinematics and performance measures across the pipeline. To capture the dynamic motion of individual vertebrae, biplane videoradiography is used in conjunction with corresponding bone model(s). This technique uses two synchronized radiographic units to simultaneously capture planar x-ray movies from two perspectives. In parallel, we create subject-specific bone models segmented from a CT scan. The process of shape-matching involves software that simulates a radiographic projection of the bone model – called a digitally reconstructed radiograph or DRR – onto the dynamic radiographic images. An optimization in each frame finds the closest match for the two projections to place and orient the vertebra in 3D space. The OrthoFusion Super Resolution approach was evaluated at each step along this process by quantifying key performance measures.

Each of the 4 cases (HR, Clin, RS, and SR) for each of the 4 participants was taken through the following pipeline shown in Fig. 3 for extracting segmental kinematics from biplane videoradiography. Briefly, the process includes simultaneous collection of dynamic radiographs from two views during cervical motion tasks, shape-matching the DRRs to the biplane radiographs, and computing the relative kinematics between the C4 and C5 vertebrae. The error associated with our traditional biplane videoradiography pipeline for the cervical spine kinematics is ≤ 0.49 mm and ≤ 1.80.^9^ The performance of each case was compared to our traditional high-resolution CT at multiple phases in pipeline.

#### Biplane Videoradiography

Dynamic radiographs of the cervical spine were acquired using a custom biplane videoradiography system (Imaging Systems & Services, Inc., Painesville, OH, USA) at 30 Hz with approximate imaging parameters of 160 mA, 70 kV, and 0.16 mSv/trial. Each participant performed a 3-second trial of neck exion-extension, lateral bending, and axial rotation. Post-processing of the radiographic images included undistortion and filtering (DSX Suite, C-Motion Inc., Germantown, MD, USA) and calibration (XMALab, Brown University, RI, USA).

#### Shape-matching

Shape-matching is the process by which 3-D kinematics are extracted by registering the DRRs (created from the projections of the segmented CT volumes) to the biplane radiographs. The dynamic 3-D positions and orientations of the C4 and C5 vertebrae were resolved using Autoscoper (Brown University, RI, USA), an open-source shape-matching software^10,11^. Both the DRRs and radiographs are filtered (Gaussian, sobel, etc.) to enhance features and edges. Auto-registration is performed using a particle swarm optimization technique with the inverse of the normalized cross-correlation (*NCC*) as the cost function. Manual refinement was performed when the optimization algorithm was unable to find an adequate match. The global 3-D kinematics of each bone was exported.

#### Shape-Matching Similarity

The match of the DRR to radiographs during the shape-matching process is quantified using the *NCC*. The *NCC* is a pixel-by-pixel correlation of the projection of the 3-D DRR to the 2-D radiographs. The inverse of the *NCC* is used as the optimization criteria for auto-registration by Autoscoper. A lower value for *NCC* value indicates a better fit. Because the NCC may be affected by radiograph image parameters, the *NCC* error metric was normalized frame-by-frame by the corresponding HR *NCC* for each participant and trial.


$$\text{NCCerror}=\frac{\left(NCC_{\text{case}}-NCC_{HR}\right)}{NCC_{HR}}$$

The mean and standard deviation of the *NCC* error was computed across all frames of each trial.

#### Relative Kinematics

Relative kinematics between vertebrae were computed using KinematicsToolbox v.4.7.2.^12^ Local coordinate systems were created for each vertebra by digitizing anatomic landmarks such that the origin is in the center of the body, x-axis points anterior, and y-axis points left, z-axis points superior.^9^ The rotations and displacements of C4 (relative to C5) were computed for each of the trials, cases, and participants.

#### Relative Kinematics Accuracy

The relative kinematics accuracy is represented by two scalar values: the orientation error and position error. The orientation error is the distance between quaternion rotations^13^ of the case, p, and HR, q, defined as:

$$=2{\text{cos}}^{-1}\left(\left|\langle p,q\rangle \right|\right)$$

$$\langle p,q\rangle =p_{1}q_{1}+p_{2}q_{2}+p_{3}q_{3}+p_{4}q_{4}$$

The position error was computed as the Euclidean distance between the relative C4–C5 displacements of the case and HR. The mean and variability (standard deviation) of the orientation and position error were computed across each trial.

### Statistics

ANOVA was used to determine groupwise differences for each outcome measure, followed by appropriate post-hoc comparisons. Normality was assessed and vertebral levels were treated as independent. A one-way repeated measures ANOVA was conducted for measures that are not impacted by trial, i.e.., image similarity and bone morphology. A two-way ANOVA was used to examine the effect of both cases and trial for the shape-matching and relative kinematics measures.

## RESULTS

**Figure 4:**
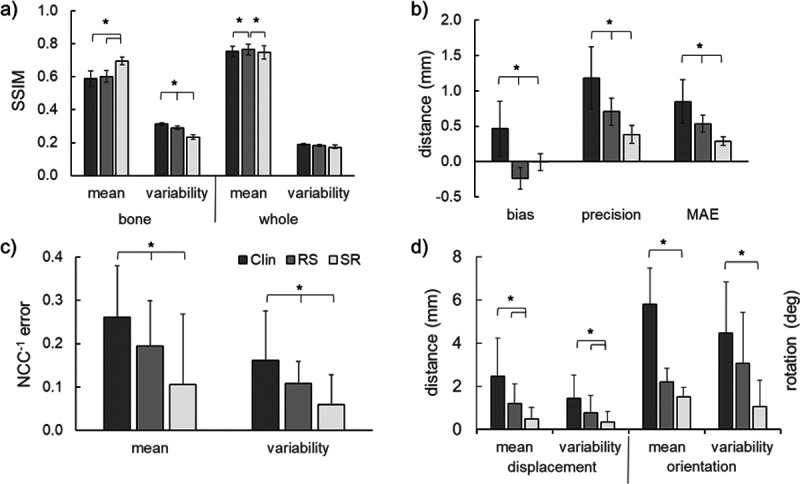
Comparisons between the simulated Clinical (Clin), Resliced (RS), and Super Resolution (SR) volumes referenced to the “ground truth” High Resolution (HR) volume in a) image similarity measures, b) bone model morphological similarity, c) shape-matching similarity, and d) relative kinematics accuracy. Error bars represent standard deviation. Asterisk indicates a Significant difference of p < 0.05 between groups. SSIM = Structural Similarity Index Measure, NCC = normalized cross-correlation coefficient.

**Figure 5:**
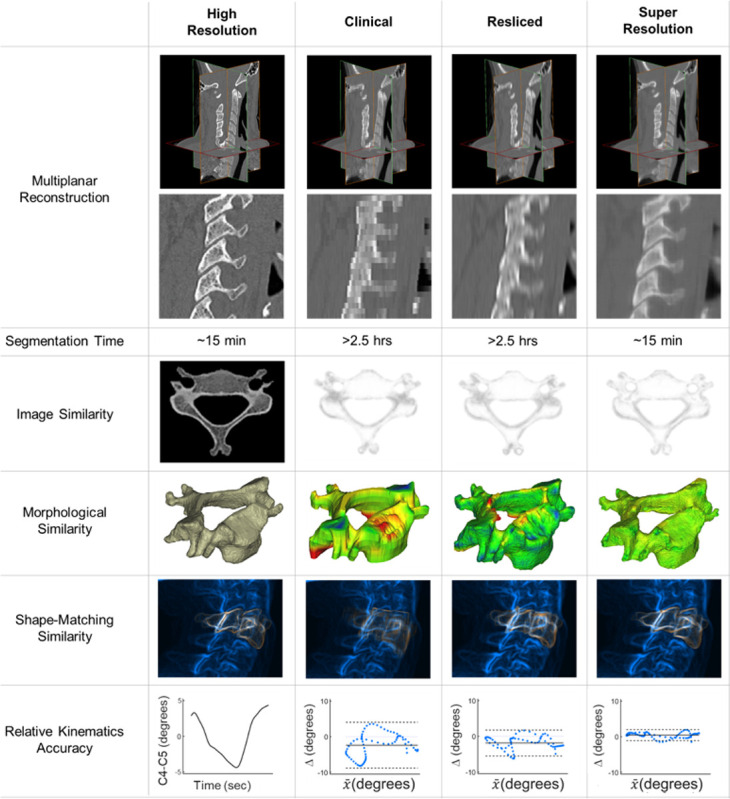
Summary Figure. Representative data illustrating the performance measures across the 4 case volumes, with the High Resolution case as the ground truth. Multiplanar Reconstructions emphasize the separation of the facet joints. Segmentation time was substantially less for the High Resolution and Super Resolution cases. Image similarity was improved with the Super Resolution approach, depicted from local SSIM maps where white indicates greater similarity. These improved performances over the Clinical and Resliced volumes yielded a greater morphological similarity and ultimately, for our applications purpose, the Super Resolution Digitally Reconstructed Radiograph (DRR) exhibited better performance for shape-matching similarity and relative kinematics accuracy.

The results are reported in Fig. 4, with representative examples in Fig. 5.

### Image Similarity

The *SSIM* of the masked volumes takes both the internal structure and the segmentation of the bone into account. The mean masked *SSIM* of the SR volume (0.696 ± 0.024) was statistically greater than the Clin (0.589 ± 0.047; p = 0.001) and RS (0.602 ± 0.037; p = 0.001) volumes. Similarly, the SR volume had significantly less *SSIM* variability (0.2 ± 0.014) than the Clin (0.313 ± 0.009; p < 0.001) and RS (0.290 ± 0.011; p < 0.001) masked volumes. The RS volume also had less variability than the Clin volume (p = 0.001).

The *SSIM* of the whole CT volumes represents the structural similarity of the non-segmented CT volumes. The mean *SSIM* of the whole RS volume (0.766 ± 0.033) was statistically higher than SR (0.747 ± 0.031, p = 0.018) and the Clin volume (0.753 ± 0.031, p < 0.001). The *SSIM* variability was not statistically different between the cases. The *PSNR* of the whole volumes were also not statistically different.

### Segmentation Time

Segmentation of each vertebra was successfully completed with the automated “CT Bone Wizard” tool in Mimics (v23) for the HR and OrthoFusion (SR) volumes with minimal need for manual refinement. This resulted in a segmentation time of approximately 15 minutes per vertebra. Manual segmentation was required for the Clin and RS vertebrae due to poor distinction between bones (primarily facet joints). This resulted in a segmentation time of approximately 2.5 hours per vertebra.

### Morphological Similarity

Significant differences between cases were found for bias, precision, and MAE distances of the 3-D bone geometry. Bias: The SR bone models (−0.01 ± 0.12 mm) had significantly less bias than the Clin (0.47 ± 0.39 mm; p = 0.017) and RS (−0.24 ± 0.15 mm; p = 0.031) bone models. Precision: The SR (0.38 ± 0.13 mm) bone models were significantly more precise than the RS (0.71 ± 0.19 mm; p = 0.003) or Clin (1.18 ± 0.44 mm; p = 0.005) bone models. MAE: The MAE of the SR bone models (0.29 ± 0.06 mm) were significantly less than the Clin (0.85 ± 0.31 mm; p = 0.002) and RS (0.54 ± 0.12; p = 0.001) bone models.

### Shape-Matching Similarity

The normalized cross-correlation coefficient (*NCC*) is the measure of pixel-by-pixel similarity of the shape-matched DRR and biplane radiographs. The *NCC* error is the normalized difference between the case and HR *NCC* values at each frame of the trial. Within-subjects effects of both trial and case were found to be Significant for the normalized *NCC* error measures. A Significant trial effect was observed such that the lateral bending trials had a significantly lower (better matched) mean *NCC* error than the axial rotation (p = 0.006) and flexion-extension (p = 0.001) trials. A Significant case effect was observed such that the mean *NCC* error for the SR cases (0.106 ± 0.162) were significantly lower than the Clin (0.261 ± 0.119; p = 0.002) and RS (0.194 ± 0.105; p = 0.024) cases. A Significant trial effect was observed such that the LB trials had significantly less variability in *NCC* error than the FE trials (p = 0.042). A Significant case effect was observed such that the *NCC* error variability for the SR cases (0.060 ± 0.014) was significantly lower than that of the Clin (0.162 ± 0.023; p = 0.002) or RS (0.109 ± 0.010; p = 0.005).

### Relative Kinematics Accuracy

The relative kinematics of the C4 to C5 vertebrae were compared between the HR and cases to determine kinematics accuracy. Significant within-subjects effects of case, but not trial, were found for the kinematics measures. The SR case produced relative kinematics with the least orientation and displacement error. The mean orientation error for the SR case (1.7 ± 1.5 deg) was significantly lower than the Clin (8.0 ± 5.8 deg; p = 0.019) and approaching significance for RS (3.8 ± 2.2 deg; p = 0.053) case. Similarly, the variability of the orientation error for the SR case (1.1 ± 1.2 deg) was significantly lower than the Clin (4.5 ± 2.3 deg; p = 0.004) and approaching significance for RS (3.1 ± 2.4 deg; p = 0.061) case. The mean displacement error for the SR case (0.5 ± 0.2 mm) was significantly lower than that of the Clin (2.5 ± 0.5 mm) and RS (1.2 ± 0.3 mm) cases. The variability of displacement error for the SR case (0.4 ± 0.49 mm) was significantly lower than the Clin (1.4 ± 1.1 mm; p = 0.014) and RS (0.8 ± 0.8 mm; p = 0.026) cases.

## DISCUSSION

We designed, implemented, and evaluated OrthoFusion super-resolution technique to increase the spatial resolution of previously acquired low-resolution CT volumes. OrthoFusion significantly improved segmentation time, the structural similarity of the bone images, and the accuracy of bone model geometry. Specific to our biplane videoradiography application, OrthoFusion improved the DRR to radiograph similarity during shape-matching and the accuracy of the relative C4–C5 kinematics. Ultimately, these improvements enable us to use previously acquired clinical CT scans from participant EMR instead of acquiring additional CT imaging as part of our studies, reducing radiation exposure, time, and cost.

Super-resolution has utility well beyond our specific biplane videoradiography application. The abundance of imaging in electronic medical records is an untapped opportunity for retrospective studies that were previously not possible due to poor through-plane resolution. Super-resolution can benefit applications requiring accurate and high resolution multiplanar reconstruction, segmentation, DRRs, and 3-D geometries. To our knowledge, currently available imaging software applications do not provide a tool that increases spatial resolution from multiple low-resolution volumes. Many super-resolution techniques have been reported in the medical imaging literature, often with the goal of exceeding hardware resolution and decreasing noise^2^ However, these algorithms are complex and challenging to implement for a typical researcher or clinician. Machine learning algorithms offer another approach, however they require training on large datasets which are not always feasible.^5,14,15^ Morphology morphing algorithms in conjunction with biplane slot scanners offer a low-dose approach to generate bone models, but are limited based on target specific geometries and do not yield a volumetric imaging dataset.^16^ OrthoFusion is an easily implemented technique that is generalizable to any CT imaging target and does not require training. Further development of the OrthoFusion algorithm by integrating filtering and iterative algorithms will provide new possibilities for the use of the large dataset of clinical imaging in EMR.

There are several limitations that should be considered in the interpretation of this study. First, we applied OrthoFusion super-resolution to our specific application. The benefits of improving spatial resolution for bone segmentation are generalizable, but performance should be assessed considering the requirements of new applications. Second, the processing and analysis involved in our biplane videoradiography pipeline requires Significant user interaction and the cases were visibly identifiable, making blinding impossible. Finally, OrthoFusion uses a simple voxel-by-voxel averaging algorithm that creates a blurring effect. The addition of anisotropic filtering^17^ or a spatial weighted average approach^18^ are likely to enhance the image quality.

This study demonstrates the utility of super-resolution to increase the spatial resolution of previously acquired low-resolution CT volumes. The OrthoFusion technique enabled us to use CT imaging from participant EMR for our biplane videoradiography studies, thus reducing exposure to radiation and costs associated with acquiring imaging. This straight-forward technique is easy to implement, generalizable to other applications, and allows researchers and clinicians that require high spatial resolution CT to utilize the abundance of clinical imaging in EMR.

## Data Availability

The code and datasets analyzed during this study are available in the Data Repository for U of M (https://conservancy.umn.edu/drum:specificlinkTBD).
